# Anatomy of a fumarolic system inferred from a multiphysics approach

**DOI:** 10.1038/s41598-018-25448-y

**Published:** 2018-05-15

**Authors:** Marceau Gresse, Jean Vandemeulebrouck, Svetlana Byrdina, Giovanni Chiodini, Philippe Roux, Antonio Pio Rinaldi, Marc Wathelet, Tullio Ricci, Jean Letort, Zaccaria Petrillo, Paola Tuccimei, Carlo Lucchetti, Alessandra Sciarra

**Affiliations:** 1 University Grenoble Alpes, Univ. Savoie Mont Blanc, CNRS, IRD, IFSTTAR, ISTerre, 38000 Grenoble, France; 2 Earthquake Research Institute, University of Tokyo, Tokyo, Japan; 3 Istituto Nazionale di Geofisica e Vulcanologia, Bologna, Italy; 4 Swiss Federal Institute of Technology (ETHZ), Zürich, Switzerland; 5 Istituto Nazionale di Geofisica e Vulcanologia, Roma, Italy; 6 Istituto Nazionale di Geofisica e Vulcanologia, Osservatorio Vesuviano, Napoli, Italy; 7 Università Roma Tre, Dipartimento di Scienze, Roma, Italy

## Abstract

Fumaroles are a common manifestation of volcanic activity that are associated with large emissions of gases into the atmosphere. These gases originate from the magma, and they can provide indirect and unique insights into magmatic processes. Therefore, they are extensively used to monitor and forecast eruptive activity. During their ascent, the magmatic gases interact with the rock and hydrothermal fluids, which modify their geochemical compositions. These interactions can complicate our understanding of the real volcanic dynamics and remain poorly considered. Here, we present the first complete imagery of a fumarolic plumbing system using three-dimensional electrical resistivity tomography and new acoustic noise localization. We delineate a gas reservoir that feeds the fumaroles through distinct channels. Based on this geometry, a thermodynamic model reveals that near-surface mixing between gas and condensed steam explains the distinct geochemical compositions of fumaroles that originate from the same source. Such modeling of fluid interactions will allow for the simulation of dynamic processes of magmatic degassing, which is crucial to the monitoring of volcanic unrest.

## Introduction

**Figure 1:**
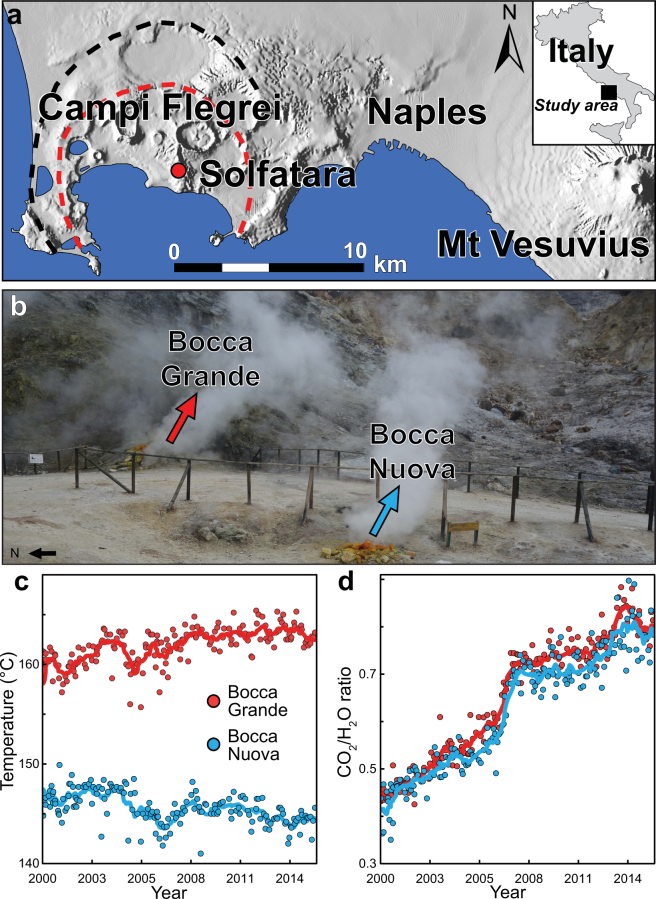
The two main fumaroles at Campi Flegrei. (**a**) Location of the Solfatara volcano. Black and red dotted lines, the two nested caldera formed by eruptions that occurred ~39 and ~15 ky ago^16–18^. This map was generated using the Esri ArcMap 10.2 software (http://www.esri.com), with the GIS database from Bechtold, *et al*.^60^. (**b**) South view of Bocca Grande and Bocca Nuova (red and blue arrows, respectively, photo Marceau Gresse, ISTerre) with the evolution of **(c)** temperature (°C) and (**d**) CO_2_/H_2_O mass ratio. Continuous line, an averaged value, performed with 2 month moving windows. The data and analytical methods used are available in Chiodini, *et al*.^4,8^. Analytical errors were assessed as lower than 3% by these authors.

Fumaroles are one of the most recurrent surface manifestations of volcanic and geothermal activities, and they release large amounts of gases into the atmosphere, mainly as H_2_O and CO_2_^1^ (Fig. 1b). They are fed by narrow permeable conduits that channel fluids toward the surface. Fumaroles can be divided in two categories, depending on their origins: geothermal and volcanic. Geothermal fumaroles are rooted in boiling hydrothermal systems, while volcanic ones are related to the magmatic system.

Studying the geochemical compositions and discharge temperatures of fumaroles is a classical and suitable method to monitor the activity of a volcano^2–6^. This can also provide understanding of the transition from quiescence to unrest, and what drives the system to a potential eruption. Indeed, eruptions are often preceded by increased gas emissions, and by variations in composition that can be monitored easily at fumarolic vents^7,8^ (Fig. 1c,d). These changes that can be perceived at the surface generally result from increased internal pressure and/or temperature, and/or changes in the composition of the magmatic reservoir. Such variations have been observed, for example, before and immediately after the onset of eruptive activity at Mount St. Helens^9^, Mount Etna^10^, Stromboli^11^, Vulcano^3^ and Asama Volcano^12^.

However, the signature composition of a fumarole does not depend only on the magmatic source, but also on other processes that occur during the ascent of the magmatic gases through the hydrothermal system^13^. In some cases, interactions between magmatic and hydrothermal fluids result in pressurization, which can lead to phreatic or phreato-magmatic eruptions^6,14^. An understanding and deconvolution of these magmatic–hydrothermal interactions is therefore required to better define the magmatic signature that is associated with volcanic unrest.

Nevertheless, assessing the unrest dynamics remains a challenging task, especially for a long-lasting active caldera^15^ with a pervasive hydrothermal system. The Campi Flegrei caldera is one such volcanic system, and was formed by two major eruptive events that occurred in the last 40 ky^16–18^ (Fig. 1a). The caldera is located in a densely populated area with ~360,000 inhabitants. It has shown clear signs of unrest since the 1950s, with an accelerating trend observed over the last decade^4^. This also represents a major concern for the three million residents of the nearby city of Naples (Italy). The renewal of the Campi Flegrei activity is driven by repeated injections of magmatic fluids into the hydrothermal system^4^. Over the last 65 years, this has led to progressive pressurization that generated a total cumulative ground uplift of ~4 m^19,20^, along with thousands of earthquakes and the enlargement of the degassing area^8,21^. This activity is mostly focused underneath the Solfatara volcano (Fig. 1a).

Numerous multidisciplinary studies have been carried out over the last decade to image this active crater. Several electrical resistivity surveys^22–26^, seismic tomography^27–29^ and petrophysical studies^30,31^ have inferred two main hydrothermal structures of the Solfatara: the liquid-dominated plume of the Fangaia mud pool, and the gas-dominated area beneath the main fumaroles. Using seismic reflection, Bruno, *et al*.^32^ defined the crucial role of faults and fractures in the control of the hydrothermal circulation. These permeable regions create an intense boiling of the aquifer, which induces significant seismic noise^22,33^ that is correlated with high soil CO_2_ degassing and temperature^23,26^.

The Solfatara volcano hosts two persistent fumaroles at the intersection of the main NW-SE and NE-SW faults, known as Bocca Grande and Bocca Nuova. These two fumaroles are ~25 m apart, and they have shown distinct temporal geochemical signatures over the past 15 years. While Bocca Grande fumarole has shown an increase in temperature, Bocca Nuova indicates the cooling of the system (Fig. 1b,c,d). The interpretation of their geochemical variations is still under debate.

Recent numerical models^4,34–36^ show that decompression of the magmatic fluids creates a vapor-dominated zone that is overlain by a steam condensation layer. This condensation can strongly impact fumarolic composition^37,38^, although its influence has never been quantified. Indeed, to date, no direct and complete imagery of a fumarolic conduit system has been achieved. Consequently, most studies have invoked large-scale assumptions about the geometry and properties of a permeable feeding structure. As a result, interactions at shallow depth between fumarole conduits and condensed steam layers have rarely been considered in volcano monitoring.

In this study, we present a multiphysics approach that allows the imaging and understanding of the shallow anatomy of a fumarolic system. First, we use previous high-resolution three-dimensional (3D) electrical resistivity tomography (ERT)^26^ to distinguish the liquid-dominated from the vapor-dominated structures. We then locate the sources of acoustic noise produced by interactions between the gas and liquid condensates to reveal the fumarole ‘plumbing’ system.

The fumarole geometry was then incorporated in a 3D multiphase flow model, which was constrained by surface observables (i.e., temperature, pressure, and CO_2_ and H_2_O fluxes). We conclude that near-surface mixing between gas and condensed steam can strongly affect both the geochemical composition and the temperature of the fumaroles. Our findings first define a multi-geophysical methodology to image the anatomy of a fumarolic system, and secondly, provide a physical model that can explain the spatial variations in the geochemical compositions of fumaroles through near-surface interactions.

## Results: The two fumarolic conduits

**Figure 2:**
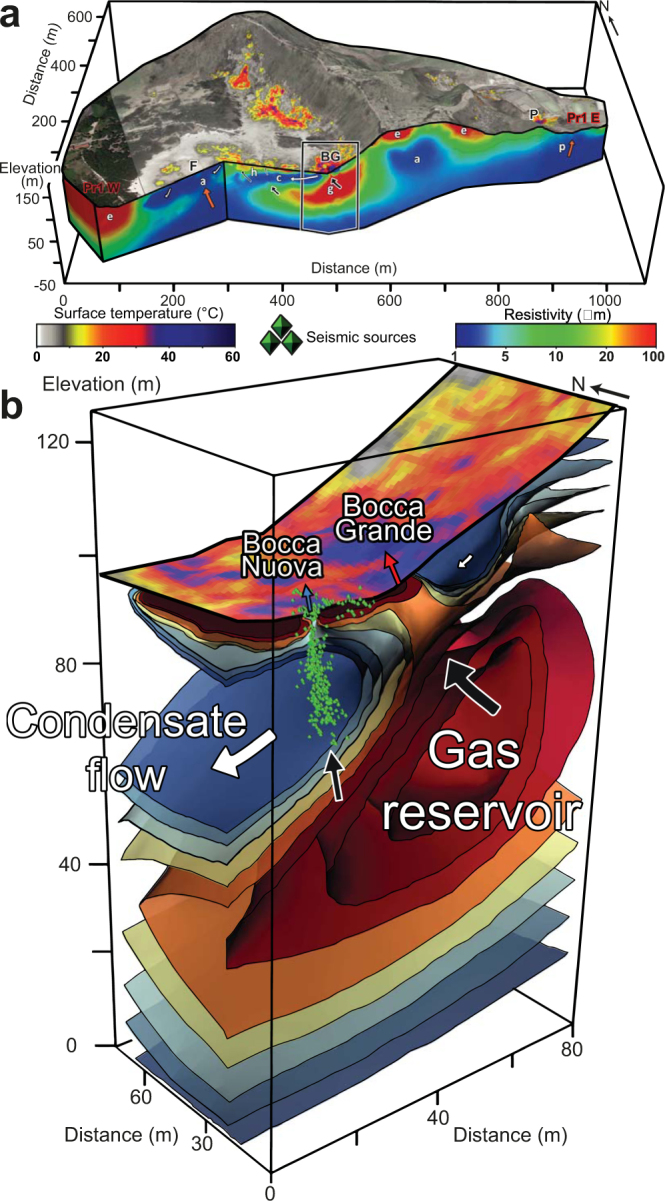
Plumbing system of the fumarolic area inferred from ERT and acoustic noise localization. (**a**) SW-NE vertical resistivity cross-section overlaid by surface temperature image (°C) crossing the Fangaia pool (F), the Bocca Grande fumarole (BG), and the Pisciarelli area (P) from Gresse, *et al*.^26^. Symbols are: g, gas-dominated reservoir; p, liquid-dominated plume; c, area of formation and circulation of condensate; e, eruptive deposits; orange arrow, convective plume; black arrow, gas flow; white arrow, liquid-dominated flow. **(b**) Three-dimensional enlargement of the electrical resistivity model beneath the fumarolic area. The gas reservoir feeds Bocca Grande through a ~10-m wide resistive channel. Seismic sources (green triangles) inferred below Bocca Nuova highlight a ~5-m-wide conduit, which channels gases from the gas reservoir toward the surface.

The resistivity model obtained from high-resolution 3D ERT by Gresse, *et al*.^26^ (see Methods, and Fig. S2 in SI) reveals the shallow fluid distribution in the fumarolic system (Figs 2 and S4; for a detailed resistivity model, see Gresse, *et al*.^26^). A gas-dominated reservoir of ~25,000 m^3^ in volume is identified ~60 meters beneath Bocca Grande and Bocca Nuova (Fig. 2a), corresponding to a closed resistive (20–40 Ωm) volume. This reservoir is connected to the Bocca Grande vent through a ~10-m-thick and at least 30-m-long resistive channel (Fig. 2b). This large gas-saturated region channels the vigorous ascent of gases. Interestingly, a similar geometry was described at Kusatsu-Shirane volcano (Japan) by Mori, *et al*.^39^, who estimated the length of the fumarole conduit (25–40 m) using acoustic resonance.

Appearing as a conductive body (1–10 Ωm), a liquid-dominated region overlies this gas reservoir. This layer is mainly formed by near-surface vapor condensation in high diffuse degassing regions on the Solfatara flanks, as reported by Gresse, *et al*.^26^. Condensate flows downslope by gravity to reach the Bocca Grande and Bocca Nuova fumarolic area, which is located at the intersection of the eastern and southern flanks of Solfatara.

**Figure 3:**
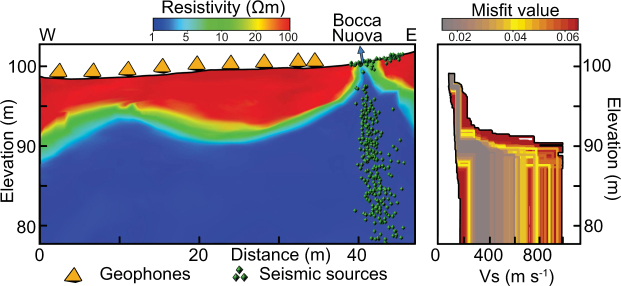
Electrical resistivity model compared with velocity models. Resistivity cross-section obtained by Gresse, *et al*.^26^ along a W-E line of nine geophones (orange triangles; see Methods and Fig. S6a in SI). The electrical resistivity model reveals a sharp contrast at ~8 m depth between a resistive (20 Ωm) and a conductive (<5 Ωm) layer. A velocity contrast is observed at the same depth, with a velocity transition from ~150 m s^−1^ to ~250 m s^−1^. The seismic sources of the Bocca Nuova conduit are represented with green triangles.

In addition to the resistivity survey, we deployed two small seismic arrays to determine whether the transfer of gases in the fumarole and their interactions with surface fluids produce any detectable signals. Coherent seismic tremors were detected in the 6 Hz to 8.5 Hz frequency band, and we applied matched field processing (MFP) techniques (see Methods, Fig. S7 in SI and Movie S1) to locate the sources of this hydrothermal tremor over time. The majority of the acoustic sources are located below Bocca Nuova, along a linear structure of ~5-m diameter and 25-m length (which corresponds to the maximum depth resolution of the survey), at an angle of 20° to the West. The velocity model obtained by dispersion curve inversion (Fig. 3; Fig. S6 in SI) indicates a sharp velocity increase at ~8-m depth, from 150 m s^−1^ to 250 m s^−1^. This location is consistent with the resistivity contrast between the 8–10-m-thick resistive layer at the surface (20–100 Ωm) and the underlying conductive body (1–10 Ωm). This disparity is likely due to the boundary between partially gas-saturated low-permeability porous rock^26,30^ and condensate layer associated with a more permeable region. This boundary does not occur in the vicinity of Bocca Nuova vent (Figs 2b and 3), where gas is escaping through a highly permeable region around the fumarolic conduit. This conductive region may be related to condensed water formed near the vent.

### Our geophysical imaging raised the following questions

1. Why do the Bocca Grande and Bocca Nuova channels have different characteristics in terms of their electrical resistivities and the occurrence of seismic tremors?
2. What is the structure defined by the tremor sources beneath the Bocca Nuova vent, and what kind of process can produce such seismic energy release?
3. How can the geophysical distinctions explain the differences in the geochemical signatures between Bocca Grande and Bocca Nuova?

To investigate these questions, we studied how the sizes and locations of these two vents can induce distinct interactions with the condensate layer, and how this layer can affect the geochemical signals of both fumaroles.

## Discussion and Numerical Modeling

The electrical resistivity model reveals a resistive gas-saturated channel that connects Bocca Grande to a gas-dominated reservoir at ~60 m in depth. However, no similar channel is inferred beneath Bocca Nuova. A calculation from synthetic resistivity models (Fig. S5 in SI) shows that a resistive gas-filled channel embedded in a conductive layer cannot be resolved in our experiments if its diameter is <5 m. In consequence, the difference in the resistivity model for the two vents is likely to originate from the greater size of Bocca Grande, which feeds a larger hot and degassing area. On the contrary, Bocca Nuova has a surface orifice a few decimeters in width.

Just below the Bocca Nuova vent, the focus of the seismic tremor sources indicates an elongated linear structure through the conductive water-saturated layer. In hydrothermal systems, high acoustic energy is produced when steam pockets cavitate in sub-cooled water^40–44^. We consider that boiling and cavitation take place around the hot Bocca Nuova conduit. The acoustic source images the boiling interface between the vertical gas up-flow within the conduit and the surrounding condensed H_2_O. This conduit is clearly distinguishable in these seismic data, although its exact location at depth can be shifted by a few meters (see the uncertainties of the model in Method and Fig. S8 in SI).

Interestingly, no significant seismic tremor sources were detected around Bocca Grande fumarole (Movie S1 in SI). The limited number of geophones cannot explain the absence of coherent sources. Indeed, several studies using the MFP method^45,46^ have shown that acoustic sources can be accurately retrieved at the edge of seismic arrays. Bocca Grande fumarole is located in an area where there are several vents that represent distributed sources (see Fig. 1b). These several sources limit the use of the MFP technique, as phase coherence between stations is no longer fulfilled. Thus, it is not possible to accurately retrieve individual events near this fumarole.

Finally, joint interpretation of the resistivity model and the acoustic source localization allow us to infer that Bocca Grande and Bocca Nuova are effectively connected to the same shallow gas reservoir through two ~30-m-long distinct conduits. The two gas paths are however different. The Bocca Grande flow does not present any localized sources, which suggests a little interaction with a liquid layer, while the Bocca Nuova gas path clearly shows interactions between the gas flow and the liquid condensate.

To explore the consistency of the geophysical model with geochemical observations at the two vents, we performed numerical simulations. The purpose was to determine whether interaction between the Bocca Nuova channel and the condensate layer can explain both its lower temperature and CO_2_/H_2_O mass ratio.

**Figure 4:**
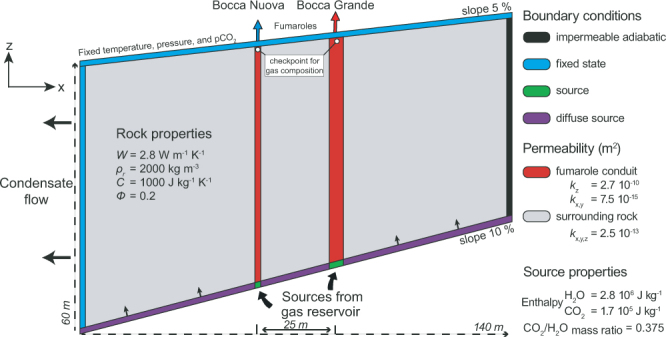
Computational domain and boundary conditions for the fumarolic system. The 3D simulation domain (140 × 140 × 60 m) is here sliced at y = 70 m to show the two fumarole conduits (in red) as ~17 m^2^ for Bocca Grande, and ~5 m^2^ for Bocca Nuova, where fluid sources are injected at the same enthalpy and CO_2_/H_2_O. The rock physical properties are: *W*, thermal conductivity; *ρ*_*r*_, rock density; *C*, specific heat; *Φ*, porosity; *k*, permeability.

**Figure 5:**
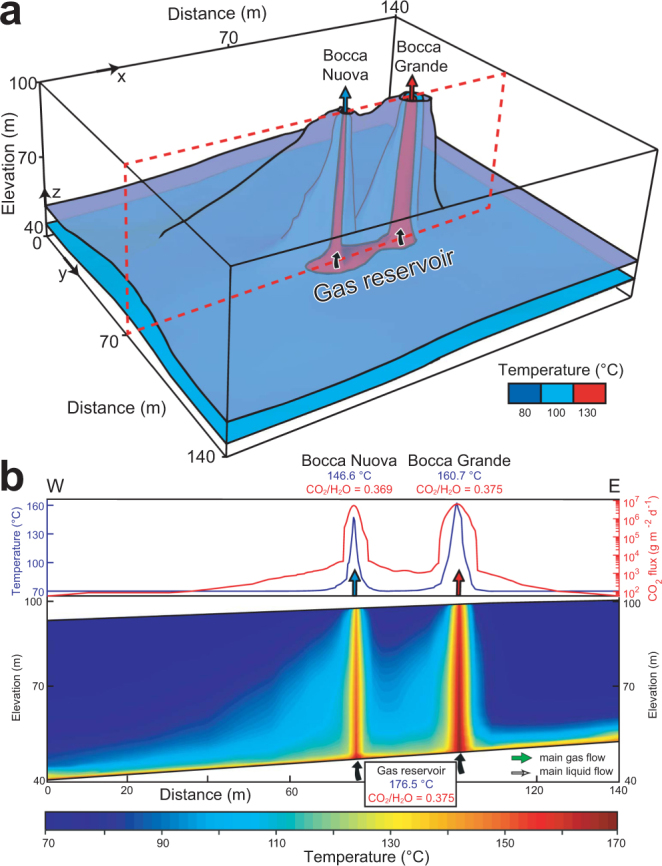
Steady-state temperature distribution model for Bocca Grande and Bocca Nuova. (**a**) Three temperature isovalues: 80, 100 and 130 °C, represented in blue, purple, and red, respectively. (**b**) W-E vertical cross-section at y = 70 m, showing the thermal structure of Bocca Grande and Bocca Nuova with surface temperature (°C) and CO_2_ flux (g m^−2^ d^−1^). Green arrows, main gas-flow; white arrows, main liquid flow.

The two-phase flow geometry obtained from the resistivity model and acoustic source location (Fig. 2b) was used to create numerical simulations of the fumarolic system using the TOUGH 2 code^47^, and to study the coupled multiphase heat and flow transfer of the CO_2_ - H_2_O mixture (see Methods). The 3D model replicates the fumarolic feeding structure to 60-m depth, with the same gas-dominated reservoir feeding both the Bocca Grande and Bocca Nuova conduits (Fig. 4). Based on knowledge of the surface fumarolic temperature, pressure, and composition, we consider that the gas in this reservoir is at hydrostatic pressure (6 bars), and contains a CO_2_ and H_2_O mixture with mass ratio 0.375 and an enthalpy of 1.7 × 10^5^ J kg^−1^ CO_2_ and 2.8 × 10^6^ J kg^−1^ respectively (Figs 4 and 5).

To reproduce the observations at the vents (CO_2_ and H_2_O flux rates and temperature^48^), the gas reservoir influx to the conduit is constrained from numerical simulation that invokes rock properties obtained from field measurements (Text S1 in SI) and are consistent with literature values^34,36,49–51^. In addition, we apply boundary conditions to replicate the upper part of the gas reservoir, at ~158 °C, and simulate the condensed water that flows westward across the conduits of the two fumaroles. Green arrows, main gas-flow; white arrows

The model of the fumarolic feeding structure shows that Bocca Grande releases ~129 tons and ~345 tons of CO_2_ and H_2_O, respectively, per day at 160.7 °C, while Bocca Nuova releases ~38 tons and ~103 tons of CO_2_ and H_2_O, respectively, per day at 146.6 °C (Figs 1c and 4). The rising mixture of CO_2_ and H_2_O creates two gas-saturated regions within the Bocca Grande and Bocca Nuova conduits, with slight overpressure (+1 × 10^4^ Pa) in their upper parts (Fig. S10 in SI). The composition of Bocca Grande is the same as that of the gas reservoir, with a CO_2_/H_2_O mass ratio of 0.375, whereas for Bocca Nuova the ratio is lower, at 0.369. The temperature differences are due to the condensed H_2_O that enters the Bocca Nuova conduit, decreasing its CO_2_/H_2_O mass ratio by ~2% and its temperature by ~9% (Fig. 5 and Fig. S11 in SI). The interactions between the hydrothermal fluids and the shallow condensate or meteoric water affects the temperature more than the CO_2_/H_2_O mass ratio due to the high latent heat of H_2_O. The higher H_2_S and CH_4_ concentration in Bocca Grande compared to Bocca Nuova (Fig. S1 in SI) might reflect near-surface oxidation processes at Bocca Nuova that are favored by the addition of shallow H_2_O containing atmospheric oxygen^38,52^_._ The systematically higher δD for Bocca Grande with respect to Bocca Nuova (Fig. S1 in SI) suggest that the shallow water enters the Bocca Nuova fumarolic channels mainly as steam, which is depleted in deuterium due to fractionation, rather than as in the liquid phase. The negligible interactions with the surrounding condensed water at Bocca Grande can be explained by its higher degassing flux (around three-fold that of Bocca Nuova), the larger radius of its vent, and its higher elevation (the pressure at the base of the Bocca Grande conduit is a little higher than the hydrostatic pressure, while at Bocca Nuova it is a little below the hydrostatic pressure).

**Figure 6:**
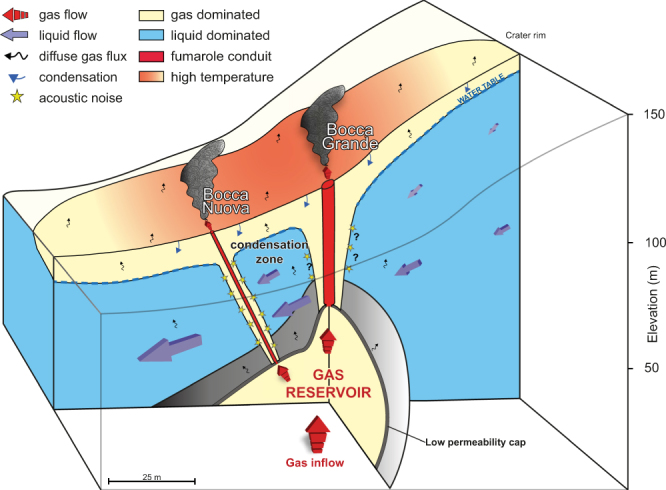
Conceptual model of a fumarolic feeding structure. Gas is stored in a shallow reservoir beneath of a low permeability cap, and directly released through two permeable conduits toward the fumarole vents. Due to atmospheric cooling and topography effects, part of the direct and diffuse degassing condenses and flows laterally. In the vicinity of the fumarole conduit, steam pockets cavitate when they encounter the cooled water flow, which produces acoustic noise (yellow stars).

These data indicate that shallow mixing can significantly influence the temperature and geochemical properties of fumarole emissions. Furthermore, they provide a conceptual model of the anatomy of a fumarole that can now be transposed to other fumarolic fields (Fig. 6).

## Conclusions

The plumbing system of the main fumarolic region of Campi Flegrei is inferred here for the first time through a combination of two geophysical approaches: high-resolution 3D ERT and acoustic noise localization. We highlight a shallow gas reservoir that feeds the two main fumaroles within the Solfatara crater, known as Bocca Grande and Bocca Nuova, through two separate 30-m-long conduits. This geophysical information is integrated within a two-phase flow model of the CO_2_–H_2_O mixture. This model accurately reproduces the fluid-flow structure and geochemical observables recorded for the fumaroles. We define a region where the H_2_O condensate and the gas up-flow mix and interact in the Bocca Nuova conduit. This interaction results in a temperature drop and a change in the CO_2_/H_2_O mass ratio at the Bocca Nuova vent. Communication between a gas-saturated conduit and condensed water is a complex mechanism that depends on the properties of both the conduit and the gas sources, and also on the dynamics of fluid circulation in the volcanic edifice. The most vigorous fumarole, Bocca Grande, is not affected by this shallow water mixing, and therefore constitutes a more relevant indicator of the volcanic unrest activity at Campi Flegrei. We reveal that it is not necessary to invoke complex mechanisms that involve magmatic components with different CO_2_/H_2_O ratios (see Fig. 3 in Moretti, *et al*.^53^) to explain the geochemical differences between these two adjacent fumaroles, as they are explained well by shallow mixing. Thus, we have here clarified the long-debated question about the origin of the distinct geochemical signatures of these two fumaroles.

## Methods

### Three-dimensional high-resolution electrical resistivity tomography

Electrical resistivity tomography is a geophysical method that is used to image shallow subsurface structures that are up to hundreds of meters beneath the surface. The process involved for each ERT measurement consists of an injection of electrical current between a pair of electrodes. The electrical field created is measured between two further electrodes. The transfer resistance is then calculated using Ohm’s law^54^ as in Equation (1):

1 $$R={\rm{\Delta }}V/I,$$

where Δ*V* is the electrical potential difference between the measurement electrodes and *I* is the injected current between the injection electrodes.

We used the surveys from Gresse, *et al*.^26^, who performed 73,987 transfer resistance measurements along 63 ERT profiles between 2008 and 2016 for the Solfatara volcano. Among these, 17 ERT profiles included 19,861 measurements taken across the fumarolic area to improve the spatial resolution of the subsoil structure (Table S1 in SI). Six radial and two circular resistivity profiles centered on the two main fumaroles, Bocca Grande and Bocca Nuova, allowed us to obtain resolution down to 1 m. The resistivity inversion was performed using the E4D code^54^, with an initial resistivity model based on an Audio-Magnetotellurics data inversion. More details about the ERT acquisition, its processing and inversion are available in Gresse, *et al*.^26^. A sensitivity map of the resistivity model is also presented in Fig. S3 in SI.

### Acoustic noise localization

The localization of continuous seismic sources in hydrothermal systems is performed using a beamforming technique called MFP^40,55,56^. For tremor activity, many overlapping events are recorded, and therefore individual time-picking algorithms are not efficient for identification and localization. MFP is based on the phase coherence of recorded time windows that consist of incoherent noise sources recorded by a geophone network^57^, and therefore is adapted to localize dominant acoustic sources with numerous individual events issuing from the same area. Using a ground-velocity model, MFP is used to create a synthetic acoustic field for each test source of a 3D grid, and to compare this with the data recorded with geophone arrays. Finally, MFP defines the probability of the source position for each element of the grid, where the highest correlations indicate the best localization.

For Solfatara, two arrays of nine vertical geophones were deployed between Bocca Grande and Bocca Nuova (Fig. S7a in SI). The acoustic noise was recorded over ~1.1 h at a sampling frequency of 250 Hz, with a cut-off frequency of 4.5 Hz. The computational MFP grid was constructed to 25 m for the X, Y and Z planes to avoid aliasing effects produced when the wavelength (λ ~ 10 m) cannot be efficiently sampled by the geophone network. We used a linear velocity gradient from 150 m s^−1^ at the surface to 250 m s^−1^ at 25 m in depth. This velocity model was derived from an active surface-wave study that was deployed to the east of Bocca Nuova (Fig. S6a in SI). The experimental dispersion curve was obtained with beamforming of 2-min-long vertical vibrations that were produced by human steps at the western end of the line. On the assumption that the fundamental Rayleigh mode was primarily excited, the curve was inverted with a neighborhood algorithm^58^. The V_s_ profiles (Fig. S6b in SI) can be reliably retrieved up to 8 m, and varied from 90 m s^−1^ to 150 m s^−1^. The theoretical ellipticity curves calculated from the V_s_ and V_p_ profiles all had a main peak that was consistent with the strong peaks observed for the experimental H/V curves (Fig. S6c in SI).

Finally, using an optimized gradient method, 4,290 source positions were obtained in 65 equal frequency intervals between 2 Hz and 14 Hz. The signal was first divided into 66 time intervals, which corresponded to 120 s of the signal, with 50% overlap. For each interval, the beamforming was processed on 20-s-long windows and with 25% overlap. To select the dominant noise sources associated with the fumarole, we plotted the optimal MFP localization *versus* the horizontal distance from the Bocca Nuova vent for each frequency. We selected the 6 Hz to 8.5 Hz band (Fig. S7b in SI) because of the higher match values combined with the maximum concentration of tremor sources.

All the spatial probabilities of these seismic sources were calculated using a classical grid search. The results presented in Fig. S8 confirm the interpretation of a small conduit connected to Bocca Nuova vent.

### Physical model of the fumaroles

Simulation of the Solfatara fumarolic system was performed using the TOUGH2 code^47^. This simulator solves coupled mass balance and energy equations for multiphase fluid flow through porous media. Fluid-flow equations are described according to Darcy Law, with a relative permeability associated to each phase (liquid, gas). Heat is transferred by conduction, convection, and latent heat effects. TOUGH2 can be applied below the critical point of water (~375 °C), which is not reached in the shallow hydrothermal system^22^ and at the fumaroles of Solfatara volcano^4^. We used the EOS2 module, because it accounts for the two main components involved in the fumarole balance: water/steam and CO_2_, including its dissolution in water.

The purpose of our physical model was to investigate the shallow hydrothermal interactions produced when a fumarole conduit crosses a H_2_O condensate flow layer, and subsequently, how those interactions will affect the gas composition and temperature of the fumarole outlet. Hence, the 3D model is based on a simplification of geophysical images. The computational domain (140 × 140 × 60 m) consists of 4,320 trapezoidal prisms with 16,194 connections. The latter was refined near the two fumaroles Bocca Grande and Bocca Nuova (Fig. S9 in SI). The channel dimensions of Bocca Grande and Bocca Nuova were inferred by acoustic noise localization and resistivity models as ~10 m and ~5 m thick, respectively. However, both methods overestimate the dimensions of the true conduits, as they imaged the whole gas-saturated region. Hence, the sizes of the conduits used in the model are assumed to be lower, and are defined arbitrarily as 50% of the gas-saturated dimension: i.e., 5 m and 2.5 m in diameter for Bocca Grande and Bocca Nuova, respectively.

We defined the same material properties for the two fumarolic conduits (Fig. 4), with a vertical permeability enhanced by two orders of magnitude with respect to the surrounding porous rock. A low permeability layer was defined at the bottom boundary, with a 10% slope to reproduce the upper limit of the gas-dominated reservoir inferred from the resistivity images. This large permeability contrast explains the sharp transition between the gas reservoir and the condensed H_2_O flowing above^59^. The model assumes homogeneous rock properties elsewhere, based on permeability measurements performed around the fumarole (Text S1 in SI) and on values from the literature^4,36^. The CO_2_ and H_2_O sources injected at the bottom of the fumarole conduits are consistent with their outlet fluxes of ~158 ± 34 t CO_2_ d^−1^ for Bocca Grande and 57.2 ± 27.2 t CO_2_ d^−1^ for Bocca Nuova^48^. We injected ~130 t CO_2_ d^−1^ and ~346 t H_2_O d^−1^ for Bocca Grande, and ~38 t CO_2_ d^−1^ and ~102 t H_2_O d^−1^ for Bocca Nuova. According to the geophysical images (Fig. 2), Bocca Grande and Bocca Nuova are supplied by the same gas reservoir at ~60 m in depth. Hence, we used the same CO_2_/H_2_O ratio for the source (Fig. 4), which was fixed at 0.375. This represents a fixed value from the year 2000, before the last unrest activity (Fig. 1d). In addition, the enthalpy was set as equal for both sources, 1.7 × 10^5^ J kg^−1^ CO_2_ and 2.8 × 10^6^ J kg^−1^ H_2_O, to inject saturated vapor at 6 bar.

The bottom boundary was also fixed, using a diffuse degassing source to reproduce the diffuse CO_2_ flux observed around the fumaroles^23^. The top boundary was fixed at atmospheric pressure conditions with a temperature of 70 °C, to reflect the thermal anomaly in this area^23,49^, and a 5% slope, to allow for the local topography. The eastern boundary is assumed to be impermeable and adiabatic, whereas the western boundary is open to allow lateral flow. Indeed, according to the resistivity model, the condensed H_2_O flow is directed westward, toward the Fangaia mud pool (Fig. 2a). The initial conditions are specified at each element with hydrostatic pressure, atmospheric CO_2_ pressure (35 Pa), and a high geothermal gradient of 1.5 °C m^−1^, consistent with the gas reservoir assumed to be at 60 m in depth.

To develop a stable physical model, we developed two steady-state approaches. The first one is the ‘building phase’, which is aimed at creating initial conditions compatible with the two-phase flow structure inferred from the resistivity model (i.e., two main fumarole conduits embedded in a two-phase shallow reservoir) and with the observed fumarole conditions (i.e., temperature, pressure, CO_2_/H_2_O mass ratio). To this end, the upper elements of the Bocca Grande and Bocca Nuova conduits were closed to fluid flow. To simulate the exchange between the upper fumarole cell and the atmosphere, we added an artificial sink that extracted the same amount of fluid injected by the source. This building phase provided a robust evaluation of the rock properties (e.g., permeability, porosity of the conduits) consistent with the surface observables, and assured convergence of the model. Indeed, we found that anisotropic permeability of the conduit was needed to limit both its lateral extent and its interaction with the condensed water (i.e., vertical permeability five orders of magnitude greater than horizontal permeability).

Using this prior knowledge, we performed the second step, the ‘steady-state phase’, using the results from the building phase as initial conditions. To correctly simulate the interactions between the fumaroles and the atmosphere, we removed the artificial Bocca Grande and Bocca Nuova sinks and fixed their respective cells at atmospheric conditions, with infinite volume.

Finally, the changes in composition were evaluated in the first element below the Bocca Grande and Bocca Nuova cells (Fig. 4). After about 2,500 years, steady-state conditions were reached, and the interactions between the condensed water and the fumaroles were consistent with the measured shift in compositions between Bocca Grande and Bocca Nuova.

### Data availability

The geophysical datasets generated and analyzed during this study are available from the corresponding author upon request. The 3D electrical resistivity forward and inverse problem was performed with the open-source code E4D (Copyright 2014, Battelle Memorial Institute), which is available online at: https://e4d.pnnl.gov/Pages/Home.aspx. The TOUGH 2 model was post-processed using TOUGH2Matlab, a free program that is available online at http://esd1.lbl.gov/research/projects/tough/licensing/free.html (written by Antonio Rinaldi). The compositions of these fumarole emissions at Campi Flegrei are available from the literature, up to December 2015^4^. Within one year the authors intend to publish all of the data from this paper and Gresse, *et al*.^26^ in the open journal Scientific Data.

## Electronic supplementary material

Supplementary Information

Movie S1
