# Supplementary material for: Anatomy of a fumarolic system inferred from a multiphysics approach
Source: Sci Rep. 2018 May 15;8:7580. doi: 10.1038/s41598-018-25448-y (PMC5954132; doi:10.1038/s41598-018-25448-y)
Supplement: Supplementary file 1 — Supplementary Information [file 41598_2018_25448_MOESM1_ESM.docx]

**Supplementary Information**

**Anatomy of a fumarolic system inferred from a multiphysics approach**

Marceau Gresse^1,2^*, Jean Vandemeulebrouck^1^, Svetlana Byrdina^1^, Giovanni Chiodini^3^, Philippe Roux^1^, Antonio Pio Rinaldi^4^, Marc Wathelet^1^, Tullio Ricci^5^, Jean Letort^1^, Zaccaria Petrillo^6^, Paola Tuccimei^7^, Carlo Lucchetti^7^ and Alessandra Sciarra^6^.

Contents of this file

Figure S1 to S11

Text S1

Table S1

Additional Supporting Information (File uploaded separately)

Movie S1


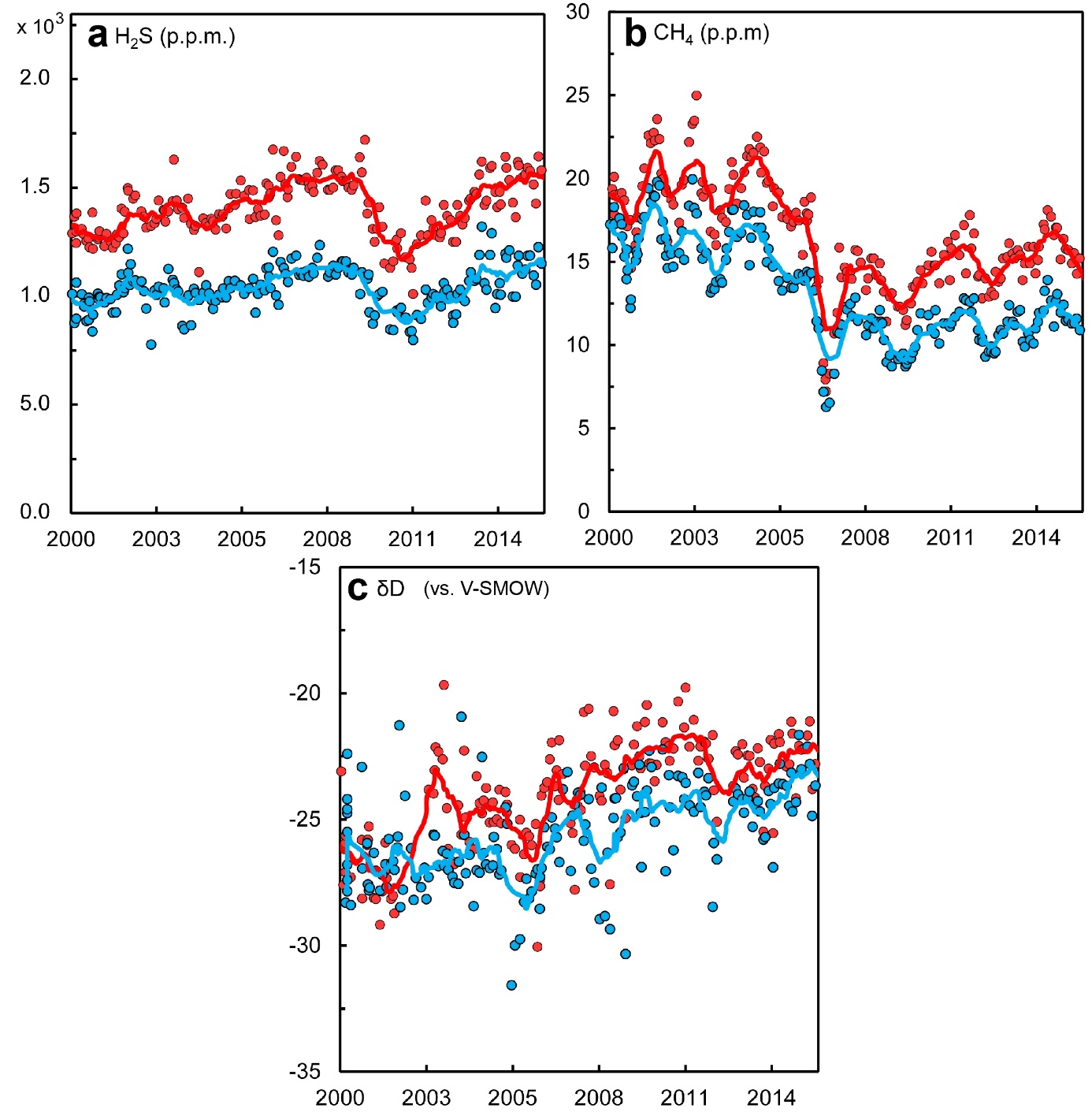


**Supplementary Figure S1. Geochemical composition of the two main fumaroles, Bocca Grande (red dots) and Bocca Nuova (blue dots) at the Campi Flegrei Caldera.** **(a)** H_2_S (p.p.m.). **(b)** CH_4_ (p.p.m.). **(c)** δD (vs. V-SMOW) since 2000. The continuous lines represents the mean values, performed with two-month moving windows. The data and analytical methods are available in [Chiodini, et al.^1^](#_ENREF_1)^,^[^2^](#_ENREF_2). Analytical errors were assessed lower than 3% by these authors.

**
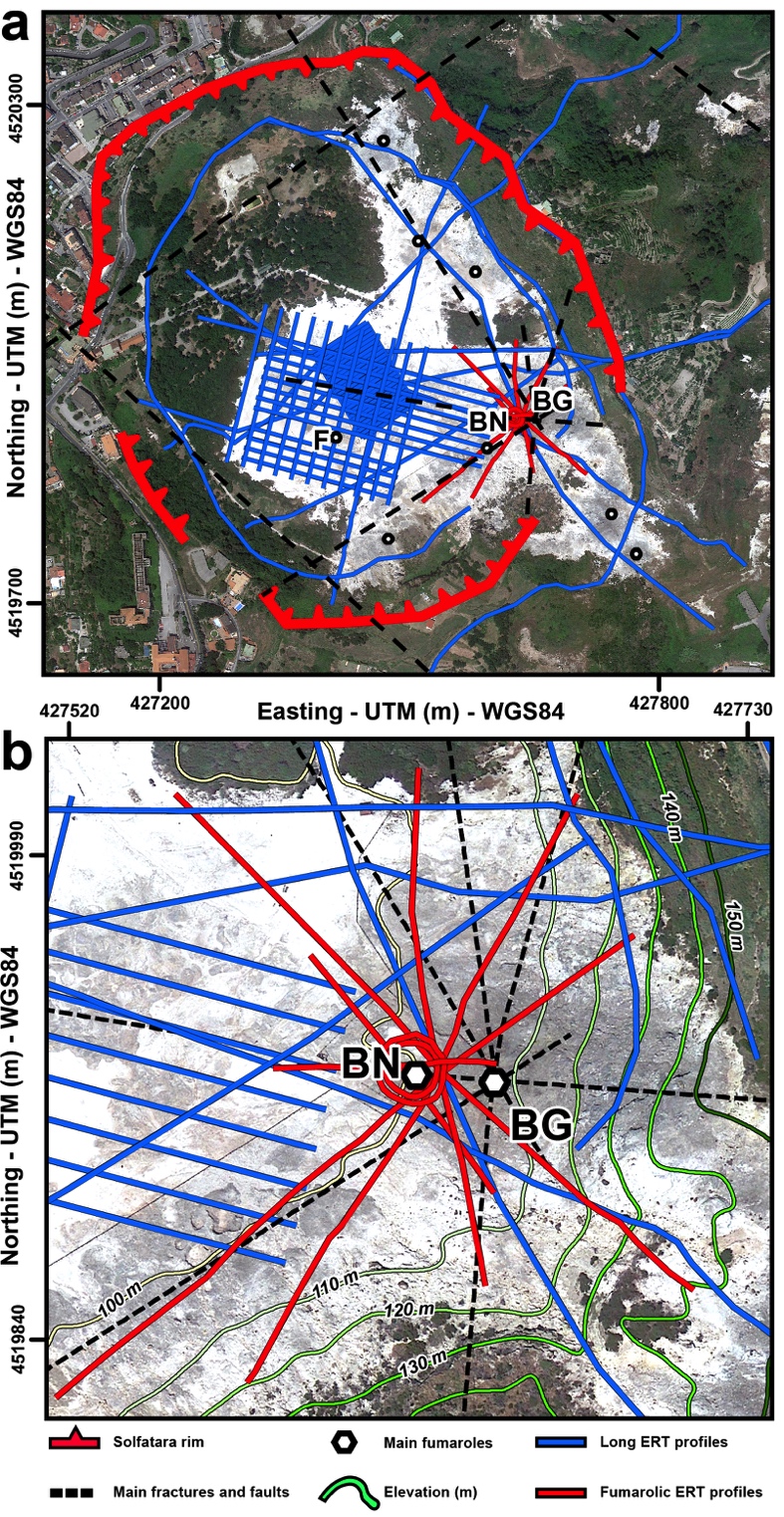
**

**Supplementary Figure S2. (a)** Map of the study area and location of the ERT profiles from modified from [Gresse, et al.^3^](#_ENREF_3). **(b)** Zoom on the ERT profiles performed around Boca Grande (BG) and Bocca Nuova (BN) fumaroles. Red lines, six radial and two circle profiles; blue lines, longer ERT profiles crossing the Solfatara crater (see [Gresse, et al.^3^](#_ENREF_3)); black circles, main vents: BG, BN fumaroles, and Fangaia mud pool (F). This map was generated using the Esri ArcMap 10.2 software (http:www.esri.com).


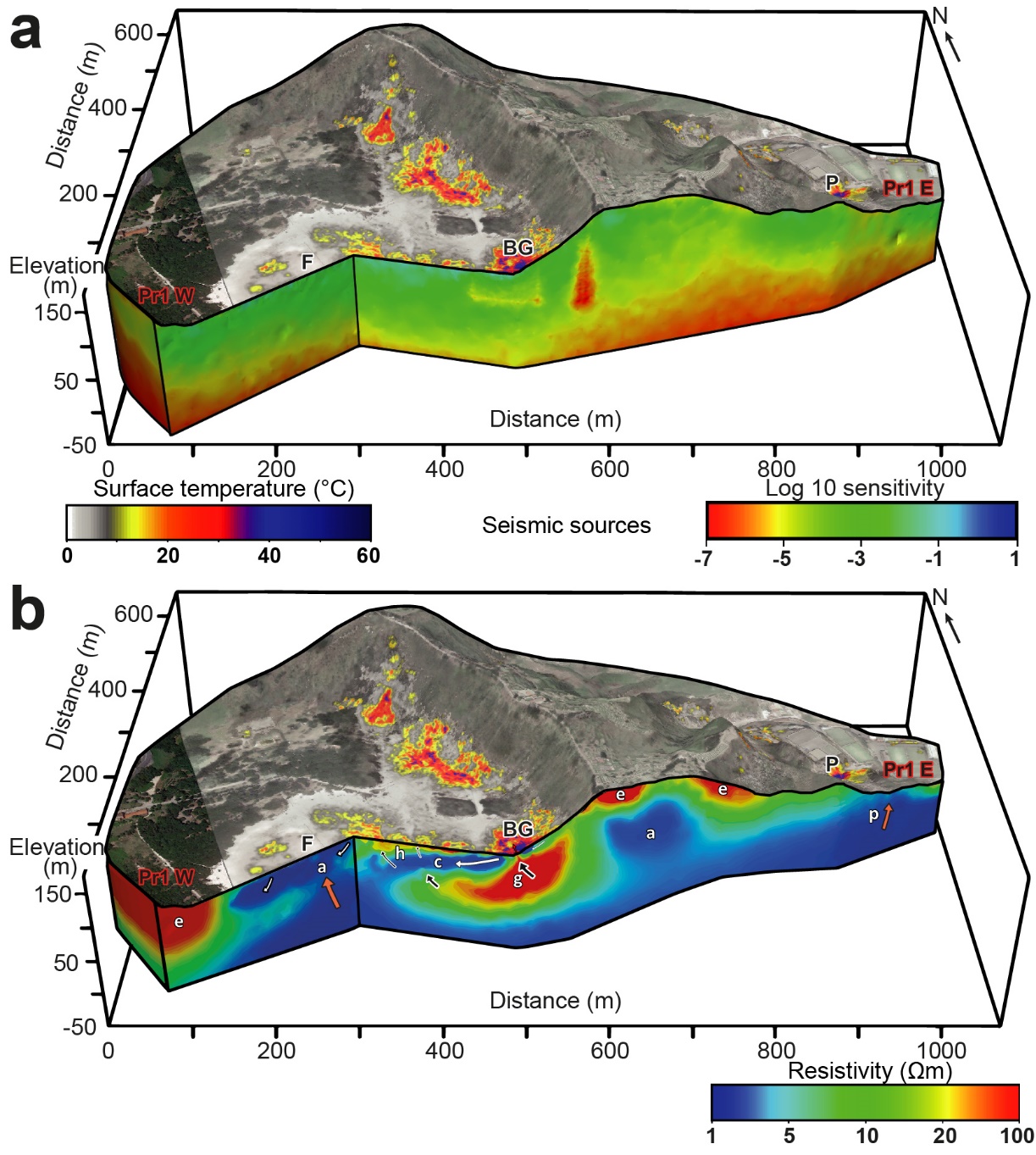


**Supplementary Figure S3.** **(a)** SW-NE vertical sensitivity of the resistivity cross-section overlaid by the surface temperature image (°C) crossing the Fangaia pool (F), the Bocca Grande fumarole (BG), and the Pisciarelli area (P); from [Gresse, et al.](#_ENREF_3)^[3](#_ENREF_3)^. The sensitivity is optimal around the Bocca Grande fumarole (down to 50 m bsl), with values higher than 10^-3^. For values below 10^-6^, the resistivity model can be considered as poorly constrained, and consequently, the resistivity cross-section **(b)** has been cut out according to this threshold. Symbols are: g, gas-dominated reservoir; p, liquid-dominated plume; c, area of formation and circulation of condensate; e, eruptive deposits; orange arrow, convective plume; black arrow, gas flow; white arrow, liquid-dominated flow.


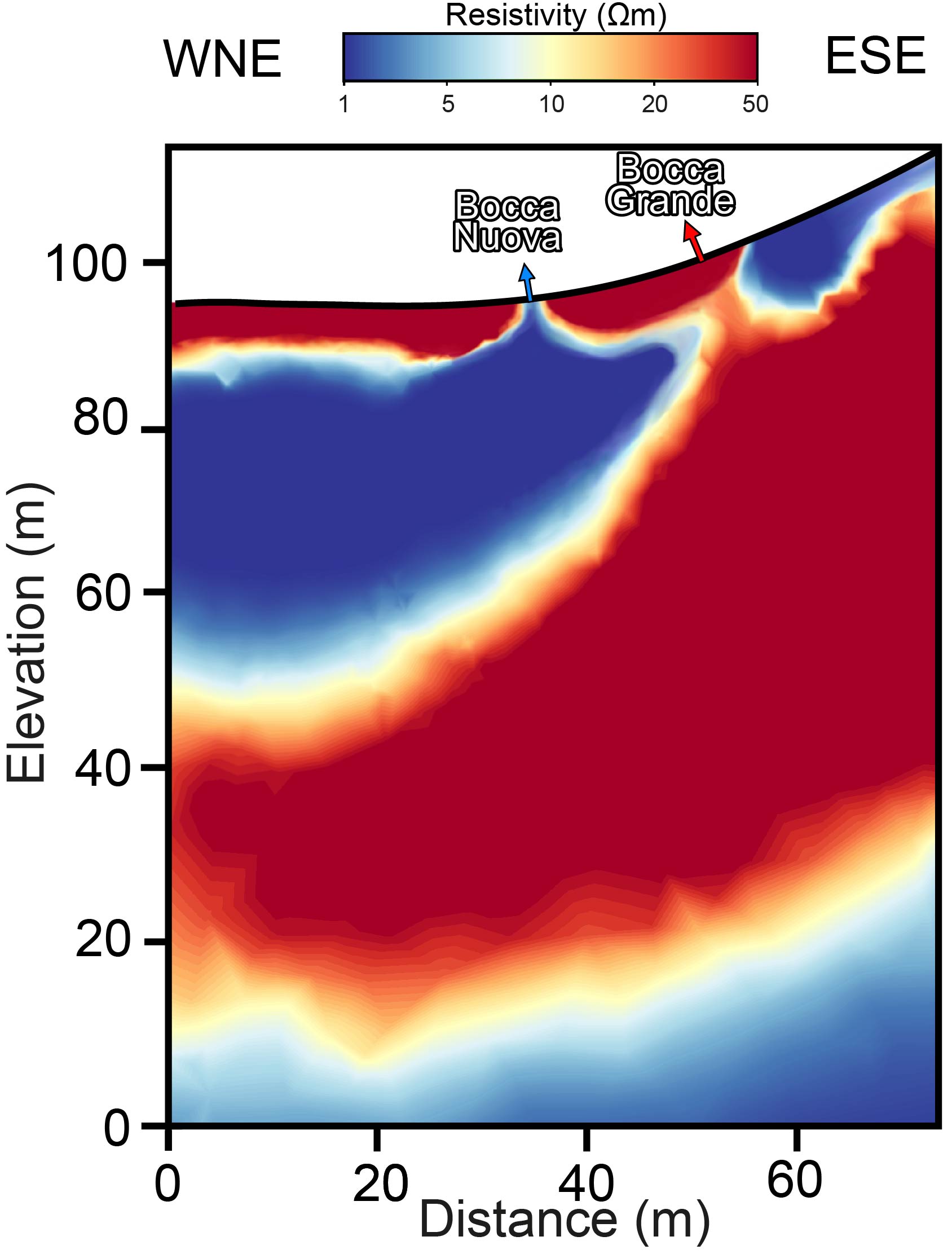


**Supplementary Figure S4. Resistivity cross-section beneath Bocca Nuova and Bocca Grande fumaroles**. This WNE-ESE cross-section is an enlargement of the resistivity model presented in Fig. 2, beneath the main fumarolic area.


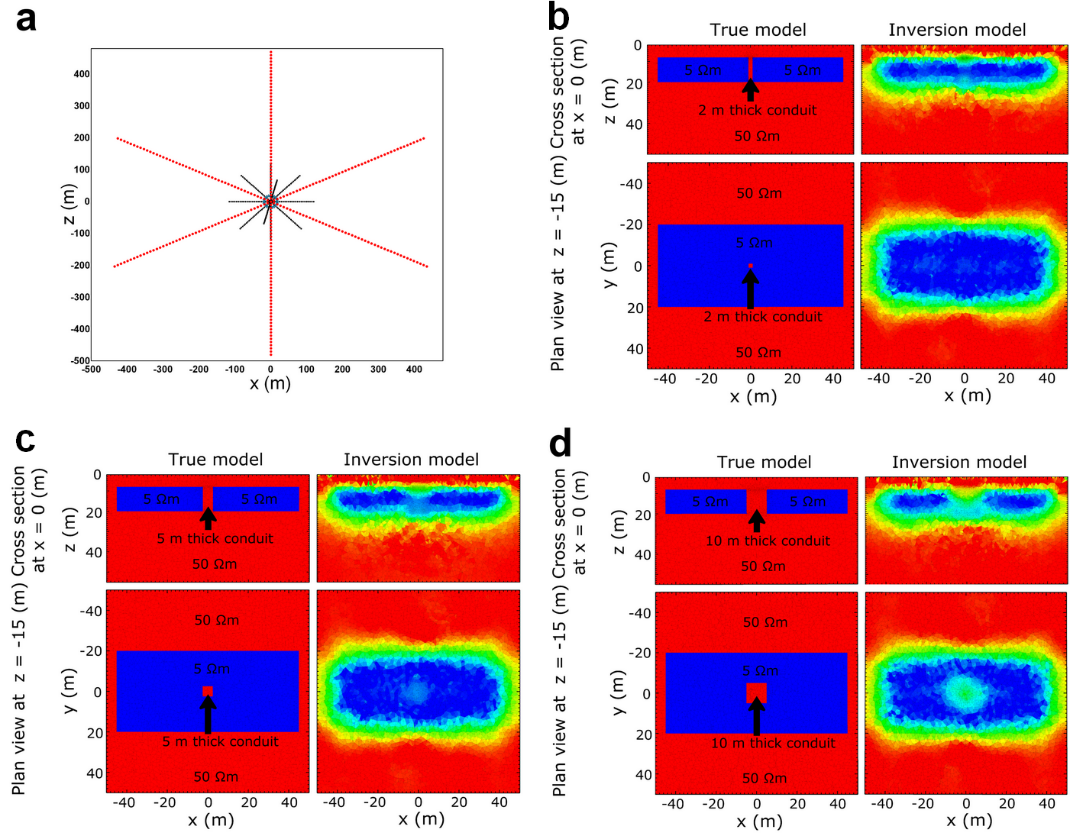
**Supplementary Figure S5. Synthetic resistivity models of a fumarolic conduit. (a)** synthetic survey created with 10,255 ERT measurements (W-S configuration) which included nine ERT profiles with 64 electrodes each. ERT profiles are centered on the fumarole conduit, to obtain similar resolution, and the survey was then carried out at the Solfatara volcano according to [Gresse, et al.^3^](#_ENREF_3). The 3D forward model is based on a true resistivity model of the fumarolic area. It is composed of a 5 Ωm layer, simulating the condensed water flow inferred in. The latter is crossed by a 50 Ωm channel that corresponds to the up-rising of gas inside the fumarole conduit. We tested three geometries for the conduit: 2, 5 and 10-m thick **(b)**, **(c)** and **(d)** respectively. The synthetic surveys were then inverted with the E4D code[^4^](#_ENREF_4) (18,617 tetrahedral elements) in 15 Gauss–Newton iterations. The convergent solution is shown with RMS <1. Resistivity inversion indicates that a resistive conduit crossing a conductive layer cannot be imaged for a conduit length <10 m (panels b, c). These data imply that the ERT method is not suitable for imaging the thin resistive fumarole here, which is surrounded by condensed water. Hence, the Bocca Nuova conduit cannot be highlighted with ERT, as the thickness of this fumarole conduit is <5 m.


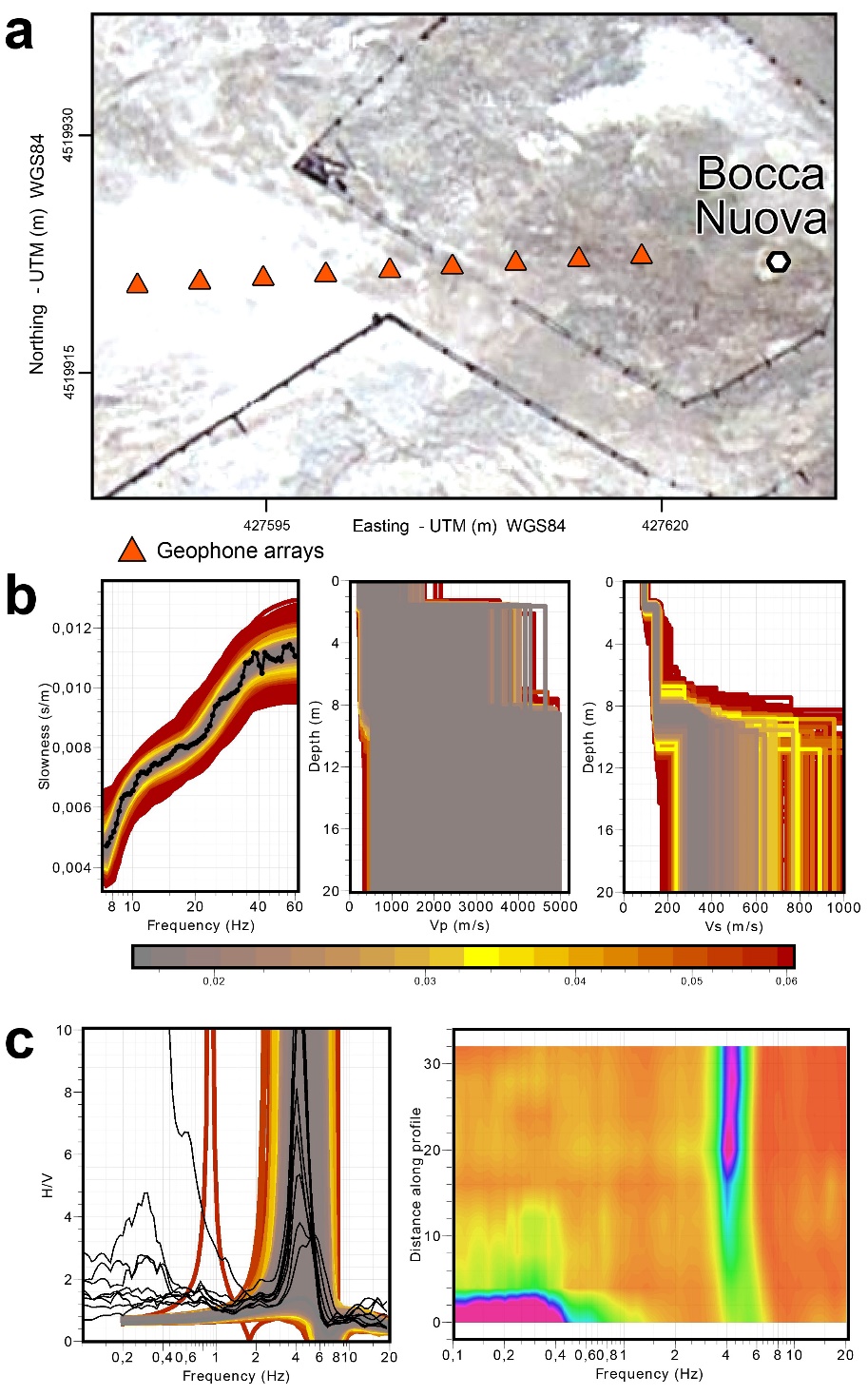


**Supplementary Figure S6.** **Active surface waves recorded with a line of geophones.** **(a)** Three-component geophone locations (orange triangles) around Bocca Nuova (black circle). This map was generated using the Esri ArcMap 10.2 software (http:www.esri.com). **(b)** Experimental (black) and inverted (colors) dispersion curves, inverted compressional (V_p_) and shear (V_s_) wave velocity profiles. Color scale shows data misfit. **(c)** Experimental spectral ratios (H/V, black) and ellipticity curves of the inverted profiles (colored). Spatial distribution of HVSR along the line. Magenta, highest amplitude. Distances are counted from the first geophone close to Bocca Nuova.

**
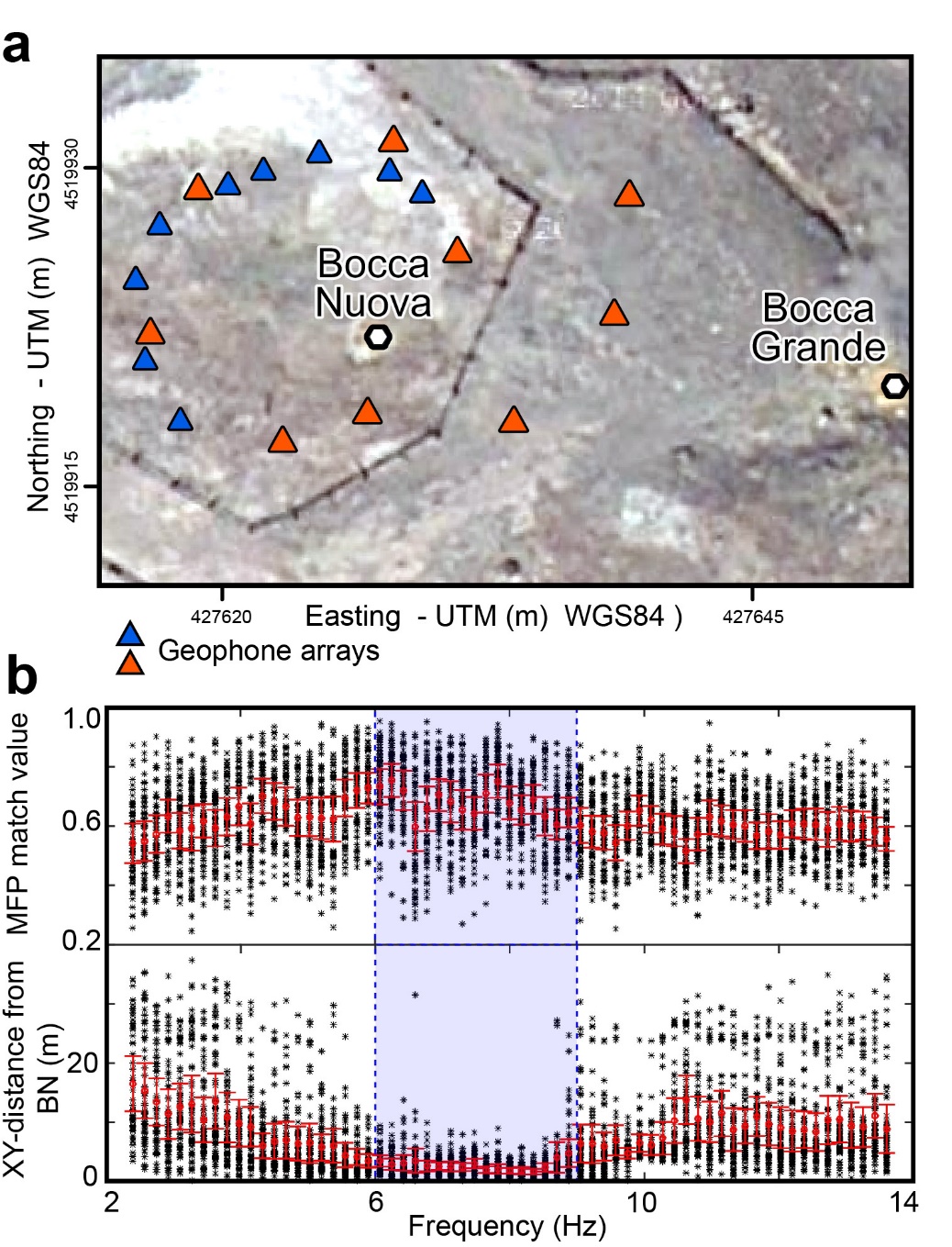
**

**Supplementary Figure S7. Geophone arrays and characteristics of the seismic sources inferred with MFP in the fumarolic area.** **(a)** Vertical geophone locations (orange and blue triangles, related to distinct configurations) around Bocca Grande and Bocca Nuova (black circles). This map was generated using the Esri ArcMap 10.2 software (http:www.esri.com). **(b)** MFP match value and horizontal distances from BN vent as a function of 65 frequency intervals between 2 Hz and14 Hz. Red, standard deviation plotted for each XY distance and MFP match value. We selected the frequencies between 6.0 Hz and 8.5 Hz (blue dotted rectangle) because they are characterized by both high match values and compact location around the BN conduit.

**
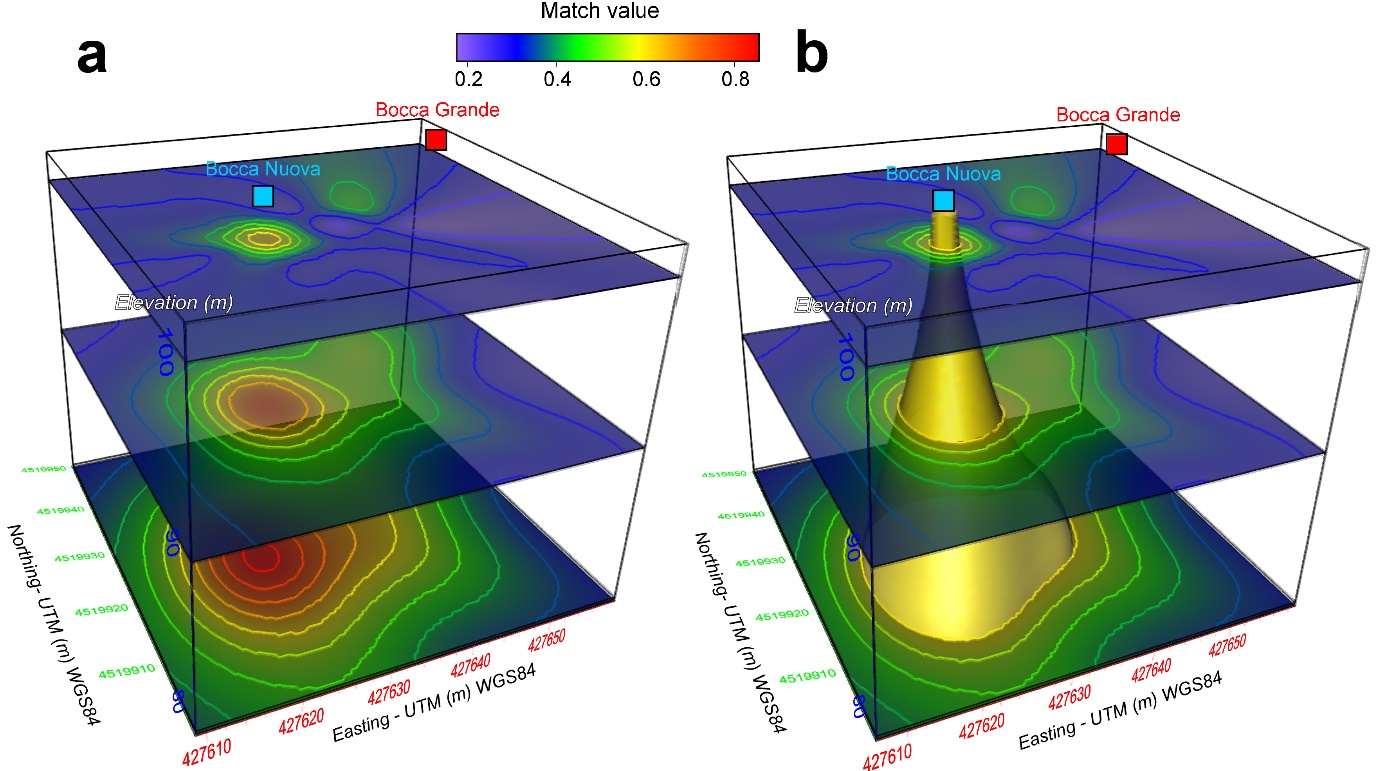
**

**Figure S8. Focal spots of the matched field processing method performed with a classical grid search approach on 15,625 1-m-sized cubic elements. (a)** Matched values of seismic sources are reported along three horizontal slices (100, 90, 80 m) beneath Bocca Nuova and Bocca Grande fumaroles for frequencies between 6.0 Hz and 8.5 Hz. **(b)** The iso-matched value of 0.6 represents the more probable area of seismic sources. The enlargement of the focal spot at depth is due to the lower resolution. Nevertheless, taking into account the maximum match values at each depth (horizontal slices), we can retrieve the channel connected to the Bocca Nuova vent.


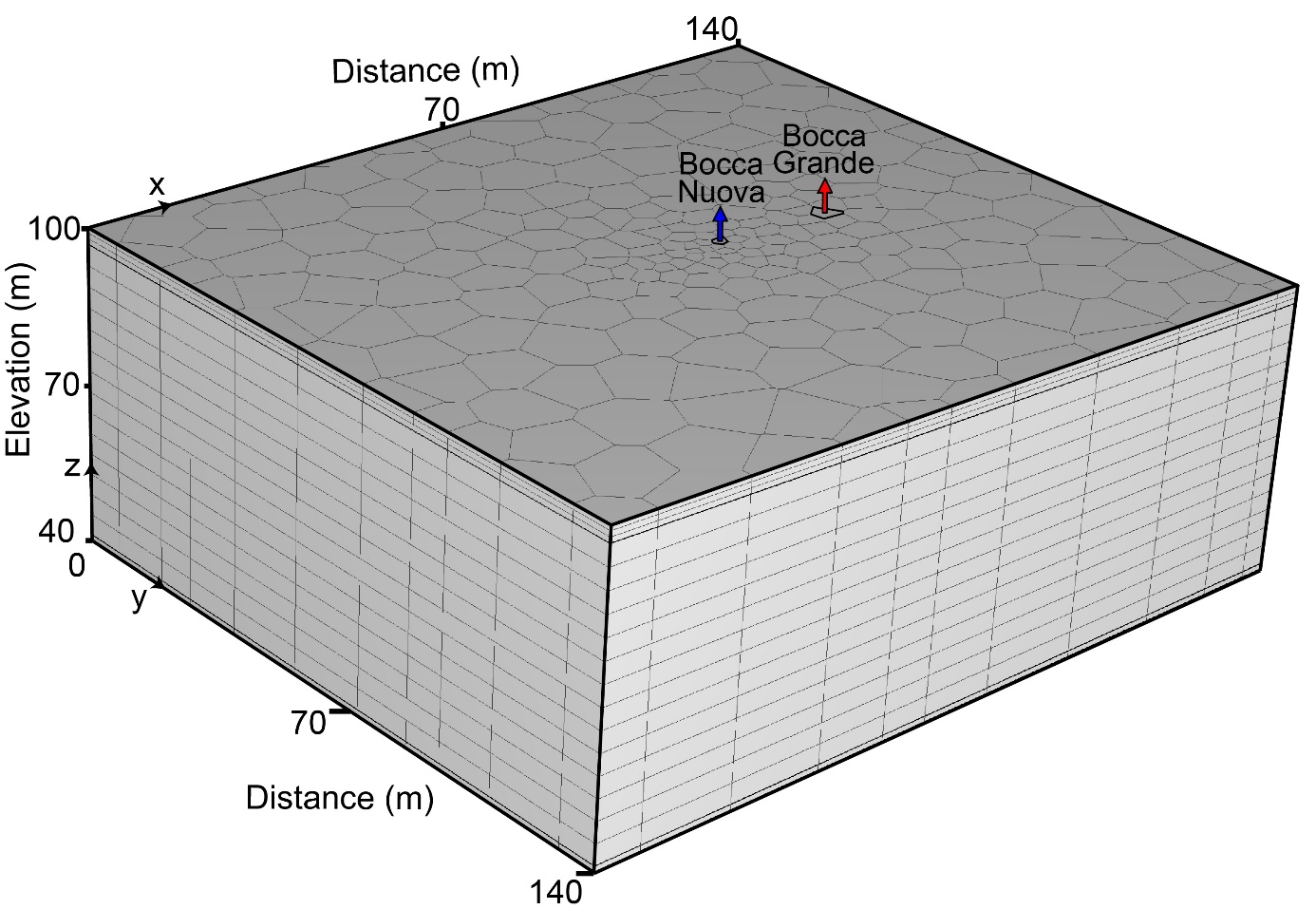
**Supplementary Figure S9. Geometry of the computational domain including the 4,320 trapezoidal prisms.** The mesh has been refined near Bocca Grande and Bocca Nuova fumaroles and in the near surface. The smallest cell has a volume of about 4.1 m^3^, while the biggest has a volume of 7.52 ×10^2^ m^3^.

**
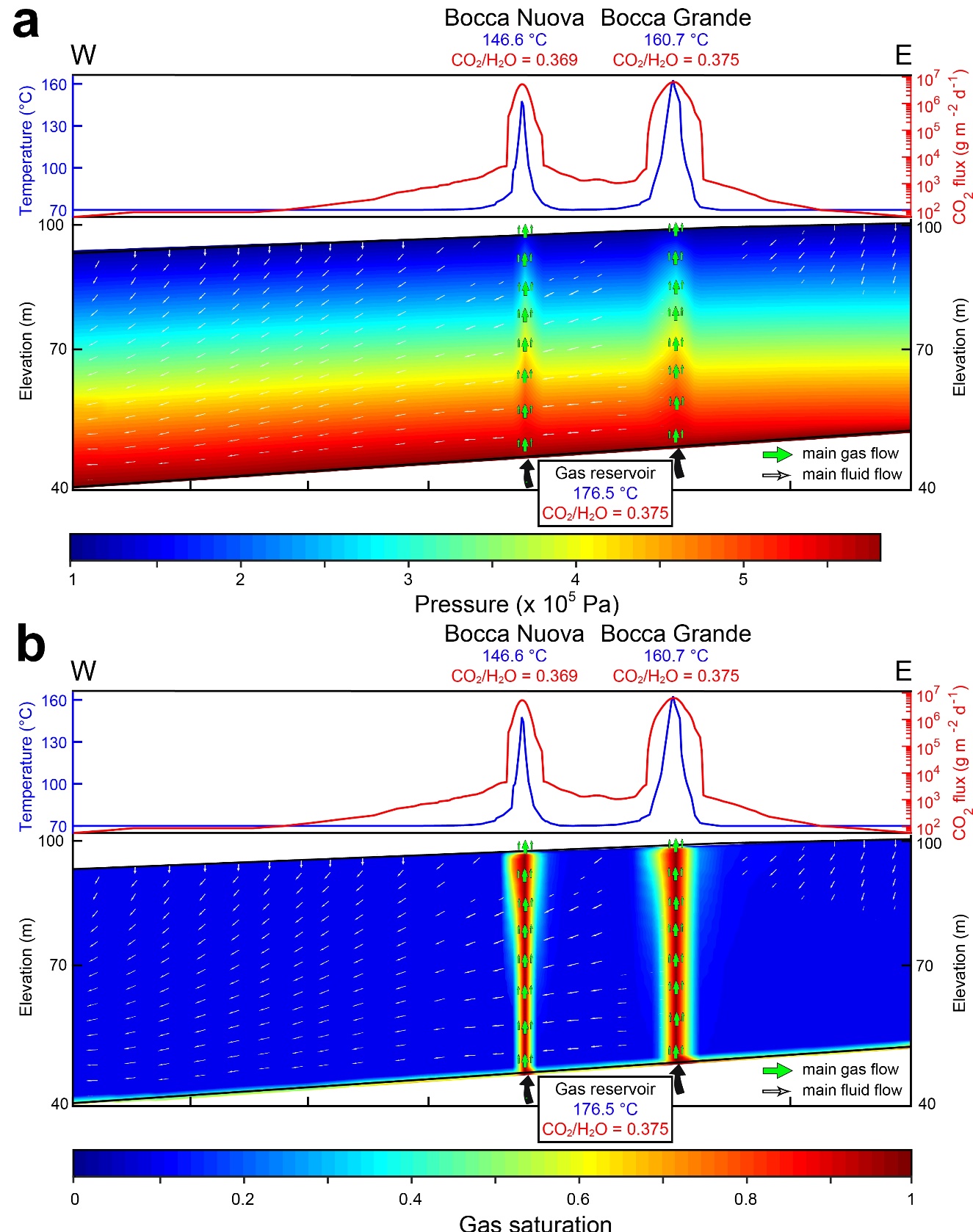
**

**a**

**Supplementary Figure S10. Pressure and gas saturation distribution of the Bocca Grande and Bocca Nuova model, at steady-state**. W-E vertical cross-section at y = 70 m, showing the pressure **(a)** and the gas saturation structure **(b)** of Bocca Grande and Bocca Nuova, with the surface temperature (°C) of the CO_2_ flux (g m^-2^ d^-1^). Green arrows, main gas-flow; white arrows, liquid flow.


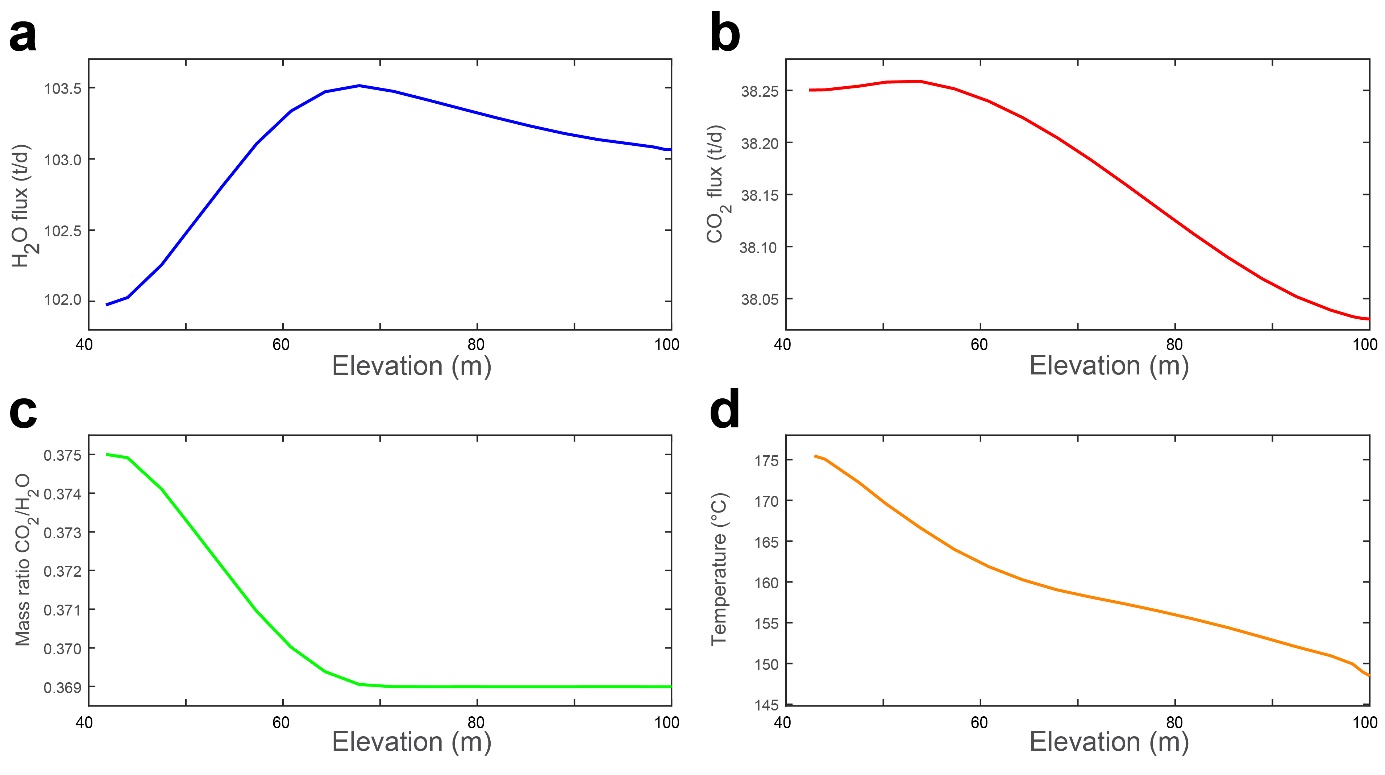


**Supplementary Figure S11. Evolution of the main multiphase flow parameters along the Bocca Nuova conduit, revealing its interaction with the condensate water**. **(a)** H_2_O flux (t d^-1^). **(b)** CO_2_ flux (t d^-1^). **(c)** CO_2_/H_2_O mass ratio. **(d)** Temperature (°C) reported along the vertical Bocca Nuova conduit (elevation in m). The increase in H_2_O flux from 102.0 to 103.5 t d^-1^ between 40 m and 70 m inside the Bocca Nuova conduit is produced by the condensate water that enters the fumarolic conduit. The latter induces a significant decrease in CO_2_/H_2_O mass ratio, as well as a reduction in the fumarole temperature.

**Supplementary Text S1.** **Method and results of soil permeability measurement.**

**Measure of soil permeability**

The gas permeability of the soil, *k* (m^2^), is calculated using Equation (S1), as derived from the Darcy Law:

$k=\frac{\mu Q}{F\cdot\Delta P}$ , (S1)

where *µ* (Pa s) is the dynamic viscosity of air (at 10 °C: *µ* = 1.75 ×10^–5^ Pa s), *Q* (m^3^ s^-1^) is the air flow through the probe, *F* (m) is the shape factor that is given as 0.149 m, and *ΔP* (Pa) is the pressure difference between the surface and the active area of the probe.

The critical point is the determination of the shape factor *F*. A solution was found in [Damkjaer and Korsbech](#_ENREF_5)^[5](#_ENREF_5)^. The resulting formula is as follows:

$F=\frac{2\pi L}{\ln\left. \left( \frac{2L \sqrt{(4D-L)/(4D+L)}}{d} \right. \right)}$, (S2),

where *L* (m) is the length of the active area, *D* (m) is the depth below the surface, and *d* (m) is the diameter of the active area.

The principle of this equipment consists of air withdrawal by means of negative pressure. Air is pumped out from the soil through a specially designed probe, with a constant surface of contact between the probe head and the soil. The constant active area is created in the head of the probe (driven into the soil to about 80 cm depth) by the extrusion of a lost tip by means of the punch wire inside the probe for an exact distance. The probes use the approximation *L* > *d*, with a shape factor *F* = 0.149 m (*L* = 50 mm, *d* = 12 mm and *D* = 825 mm).

Measurements were carried out using RADON JOK (Radon v.o.s, www.radon-vos.cz, Fig. 1 in Supplementary Text S1), which is widely used to determine soil intrinsic permeability ^[6](#_ENREF_6)^ ^[7](#_ENREF_7)^. In areas affected by high-temperature degassing, such as Solfatara (soil temperatures ranged from 20.1 to 94.5 °C), the permeability value needs to be corrected, because the air viscosity (which is directly correlated with permeability) is strongly dependent on the gas composition and temperature. The gas analysis was carried out at the Fluid Geochemistry Laboratory of INGV Rome.


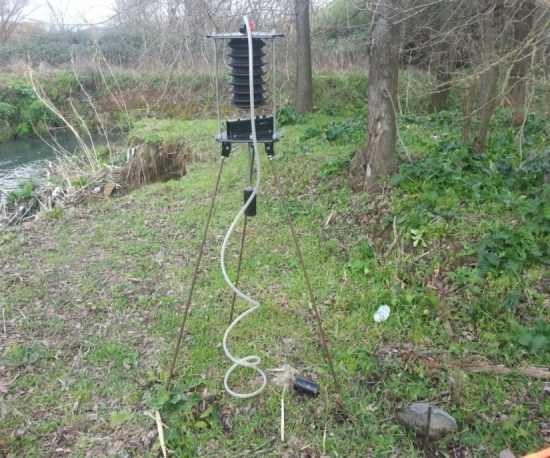


**Figure 1 (Supplementary Text S1).** RADON JOK permeameter (Photo Tullio Ricci, INGV).

**Table 1 (Supplementary Text S1):** ***In-situ* soil permeability measurements performed at Solfatara volcano.** Twenty-one measurements were carried out in May 2015 at 20 cm and 100 cm depth, using a RADON JOK Permeameter.

| **Measurement** | **UTM WGS84** | | **Permeability (m^2^)** | |
| --- | --- | --- | --- | --- |
|  | **X (m)** | **Y (m)** | **20 cm** | **100 cm** |
| 1 | 427582.8 | 4519923.3 | 7.92E-13 | 1.04E-12 |
| 2 | 427567.9 | 4519928.4 | 1.90E-12 | 1.22E-12 |
| 3 | 427455.6 | 4519968.6 | 1.11E-12 | 3.82E-13 |
| 4 | 427623.7 | 4519922.9 | 1.42E-12 | 1.82E-12 |
| 5 | 427492.5 | 4519893.1 | 2.84E-12 | 2.21E-12 |
| 6 | 427409.9 | 4519911.8 | 9.91E-14 | 1.36E-14 |
| 7 | 427630.2 | 4519980.1 | 2.11E-13 | 1.09E-12 |
| 8 | 427529.0 | 4520151.5 | 1.14E-13 | 2.29E-13 |
| 9 | 427647.8 | 4519911.2 | 1.08E-12 | 2.33E-13 |
| 10 | 427190.5 | 4519941.7 | 3.65E-13 | 2.28E-12 |
| 11 | 427384.3 | 4520015.6 | 1.19E-12 | 5.71E-13 |
| 12 | 427546.8 | 4519870.1 | 1.33E-12 | 1.36E-12 |
| 13 | 427445.7 | 4519834.3 | 1.73E-12 | 1.75E-12 |
| 14 | 427631.2 | 4520016.2 | 1.75E-13 | 2.33E-12 |
| 15 | 427599.7 | 4520025.9 | 1.47E-12 | 3.20E-12 |
| 16 | 427846.0 | 4519697.0 | 1.35E-12 | 9.79E-15 |
| 17 | 427366.0 | 4519905.0 | 2.02E-13 | 2.72E-14 |
| 18 | 427569.5 | 4519987.1 | 2.30E-12 | 9.37E-13 |
| 19 | 427458.0 | 4520221.9 | 1.39E-12 | 1.27E-12 |
| 20 | 427452.5 | 4520070.1 | 2.16E-12 | 2.35E-12 |
| 21 | 427525.6 | 4520042.5 | 7.35E-13 | 2.17E-12 |

**Selection of permeability values for the multiphase flow model**


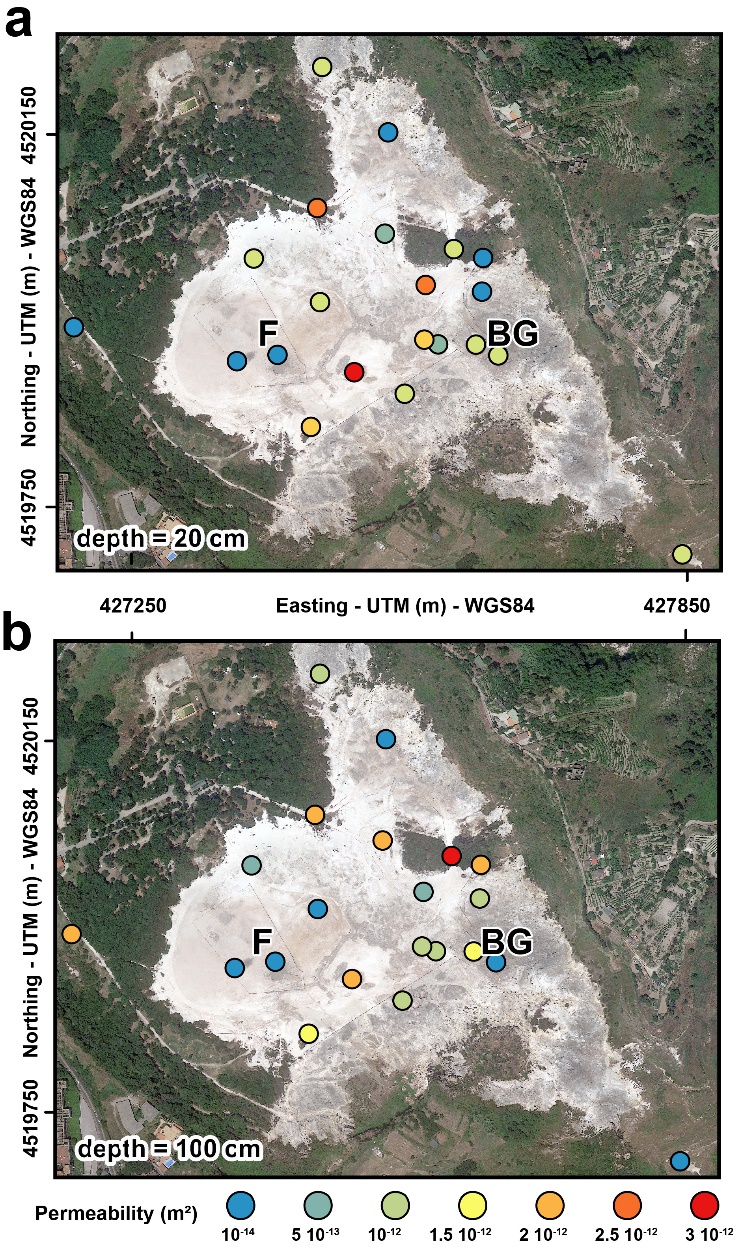
The soil permeability measurements at Solfatara ranged between 10^-12^ and 10^-14^ m² (Fig. 2 in Supplementary Text S1). A permeability of 2.5 ×10^-13^ m² was chosen for the surrounding rock of the multiphase flow model, as this represents an average value of the main altered area inside the Solfatara. This value is also consistent with tuff measurements at Campi Flegrei Caldera^[8](#_ENREF_8)^ and previous multiphase flow models associated to fumarolic highly permeable regions^[9-11](#_ENREF_9)^.

**Figure 2 (Supplementary Text S1).** Location and range of soil permeability measurements (m²) performed at a depth of 20 cm **(a)** and 100 cm **(b)** at Solfatara. Labels are: BG, Bocca Grande fumarolic area and F, Fangaia. This map was generated using the Esri ArcMap 10.2 software (http:www.esri.com).

**Supplementary Table S1. Electrical resistivity profiles crossing the fumarolic area of the Solfatara volcano**. Details of the electrodes and the array configuration collected for each electrical resistivity profile. W-S, Wenner-Schlumberger; W, Wenner; PP, pole-pole.

| **N°** | **Name** | **Date** | **Number of electrodes** | **Spacing** | **Number of raw measurements** | **Number of filtered measurements** | **Array** |
| --- | --- | --- | --- | --- | --- | --- | --- |
| 1 | ERT1 | May-2012 | 96 | 10 | 2661 | 2429 | W-S |
| 2 | ERT2 | May-2012 | 96 | 10 | 2661 | 2538 | W-S |
| 3 | ERT3 | Oct-2014 | 82 | 10 | 5083 | 1319 | W-S |
| 4 | ERT5 | Mar-2015 | 47 | 10 | 565 | 507 | W-S |
| 5 | ERT7-a | Jan-2016 | 64 | 20 | 1302 | 596 | W-S |
| 6 | ERT-fum.1 | Mar-2015 | 48 | 5 | 565 | 260 | W-S |
| 7 | ERT-fum.1 | Mar-2015 | 48 | 5 | 1128 | 1108 | PP |
| 8 | ERT-fum.2 | Mar-2015 | 48 | 5 | 565 | 248 | W-S |
| 9 | ERT-fum.2 | Mar-2015 | 50 | 5 | 1128 | 571 | PP |
| 10 | ERT-fum.3 | Mar-2015 | 48 | 5 | 565 | 404 | W-S |
| 11 | ERT-fum.3 | Mar-2015 | 50 | 5 | 1128 | 847 | PP |
| 12 | ERT-fum.4 | Mar-2015 | 48 | 5 | 565 | 560 | W-S |
| 13 | ERT-fum.4 | Mar-2015 | 50 | 5 | 1128 | 924 | PP |
| 14 | ERT-fum.5 | Mar-2015 | 48 | 2 | 565 | 553 | W-S |
| 15 | ERT-fum.6 | Jua-2016 | 24 | 3 | 84 | 78 | W |
| 16 | ERT-fum.7 | Jua-2016 | 24 | 2 | 84 | 84 | W |
| 17 | ERT-fume.8 | Jua-2016 | 24 | 3 | 84 | 77 | W |
| **Total** | | | **823** |  | **19861** | **13103** |  |

**Supplementary Movie S1. Animation of the fumarolic plumbing system inferred from the 3-D electrical resistivity model from [Gresse, et al.](#_ENREF_3)^[3](#_ENREF_3)^, superimposed with acoustic seismic sources.** The resistivity model of the Solfatara crater (Ω m) is overlaid by the surface temperature image (°C). The gas-dominated reservoir, 60 m in depth (20-40 Ω m, in red), directly feeds Bocca Grande (BG) fumarole through a ~10-m-thick resistive conduit. The camera rotates, zooms in on the fumarolic area, and shows the 4290 unfiltered seismic sources (blue-red dots). The movie finally displays selected seismic sources (6.0-8.5 Hz, green dots) and reveals the ~5-m-thick channel that connects Bocca Nuova (BN) fumarole to the gas-dominated reservoir.

**References**

1 Chiodini, G. *et al.* Magmas near the critical degassing pressure drive volcanic unrest towards a critical state. *Nature Communications* **7**, 13712, doi:10.1038/ncomms13712 (2016).

2 Chiodini, G. *et al.* Evidence of thermal-driven processes triggering the 2005–2014 unrest at Campi Flegrei caldera. *Earth and Planetary Science Letters* **414**, 58-67, doi:10.1016/j.epsl.2015.01.012 (2015).

3 Gresse, M. *et al.* 3-D electrical resistivity tomography of the Solfatara crater (Italy): implication for the multiphase flow structure of the shallow hydrothermal system. *Journal of Geophysical Research: Solid Earth*, doi:10.1002/2017JB014389 (2017).

4 Johnson, T. C., Versteeg, R. J., Ward, A., Day-Lewis, F. D. & Revil, A. Improved hydrogeophysical characterization and monitoring through parallel modeling and inversion of time-domain resistivity and induced-polarization. *Geophysics* **75**, doi:10.1190/1.347551 (2010).

5 Damkjaer, A. & Korsbech, U. A Small-Diameter Probe for In Situ Measurements of Gas Permeability of Soils. *Radiation Protection Dosimetry* **45**, 85-89, doi:10.1093/oxfordjournals.rpd.a081502 (1992).

6 Neznal, M., Matolin, M., Barnet, I. & Miksova, J. The new method for assessing the radon risk of building sites. *Czech Geological Survey Special Papers* **16** (2004).

7 Castelluccio, M., Giannella, G., Lucchetti, C., Moroni, M. & Tuccimei, P. Classification of radon hazard in urban planning focused to risk management (In Italian). *Italian Journal of Engineering Geology and Environment* **2**, 5-16, doi:10.4408/IJEGE.2012-02.O-01 (2012).

8 Vanorio, T., Prasad, M., Patella, D. & Nur, A. Ultrasonic velocity measurements in volcanic rocks: correlation with microtexture. *Geophysical Journal International* **149**, 22-36, doi:10.1046/j.0956-540x.2001.01580.x (2002).

9 Todesco, M., Rinaldi, A. P. & Bonafede, M. Modeling of unrest signals in heterogeneous hydrothermal systems. *Journal of Geophysical Research: Solid Earth* **115**, doi:10.1029/2010JB007474 (2010).

10 Rinaldi, A. P., Todesco, M., Vandemeulebrouck, J., Revil, A. & Bonafede, M. Electrical conductivity, ground displacement, gravity changes, and gas flow at Solfatara crater (Campi Flegrei caldera, Italy): Results from numerical modeling. *Journal of Volcanology and Geothermal Research* **207**, 93 - 105, doi:10.1016/j.jvolgeores.2011.07.008 (2011).

11 Jasim, A., Whitaker, F. F. & Rust, A. C. Impact of channelized flow on temperature distribution and fluid flow in restless calderas: Insight from Campi Flegrei caldera, Italy. *Journal of Volcanology and Geothermal Research* **303**, 157-174, doi:10.1016/j.jvolgeores.2015.07.029 (2015).
